# Time and amount attributes exert distinct neural influences on gain and loss evaluation

**DOI:** 10.1093/scan/nsag029

**Published:** 2026-05-06

**Authors:** Ya Zheng, Chenlu Guan, Puyu Shi

**Affiliations:** Department of Psychology, Guangzhou University, Guangzhou 510006, China; Center for Reward and Social Cognition, School of Education, Guangzhou University, Guangzhou 510006, China; Department of Psychology, Dalian Medical University, Dalian 116044, China; Department of Psychology, Dalian Medical University, Dalian 116044, China

**Keywords:** time, amount, gain–loss asymmetry, delay discounting, ERPs

## Abstract

People typically discount delayed gains more than delayed losses, reflecting a gain–loss asymmetry. This event-related potential study examined how neural representations of amount and time, two prominent attributes in delay discounting, are influenced by contextual valence (gains vs. losses) through the lens of neural dynamics. Forty participants performed a simple guessing task in which they experienced a gain context with varying reward amounts delivered at different time delays, and a loss context with varying loss amounts delivered similarly. Time and amount attributes were manipulated orthogonally and parametrically. Results showed that in the gain context, time attribute was first tracked during the reward positivity period, then integrated with linearly increasing monetary value during the P3 period, and finally processed independently from monetary value during the late positive potential period. In the loss context, time was similarly processed during the RewP period, whereas monetary value was not tracked until the late positive potential period. Additionally, a correlation between behavioral and neural delay discounting was found in the loss but not gain context. Our findings demonstrate distinct evaluative processes for time and amount attributes between gain and loss contexts, providing new insights into the gain–loss asymmetry in delay discounting.

## Introduction

Most people prefer to receive rewards sooner rather than later—a tendency known as delay discounting, which refers to the decrease in the subjective value of a reward as the delay to its receipt increases (Ainslie 1975). For example, if you won a lottery prize, you would likely want to claim it immediately rather than wait. Similarly, everyday decisions often involve negative outcomes as well as positive ones. If you received a traffic fine, you would probably prefer to postpone payment for as long as possible. Although both gains and losses diminish in subjective value over time, numerous behavioral studies have demonstrated that people typically discount delayed gains more steeply than delayed losses, a phenomenon known as the sign effect (Thaler 1981, Estle *et al.* 2006). Despite extensive behavioral evidence for this gain–loss asymmetry in delay discounting, its underlying neural mechanisms remain poorly understood (Tanaka *et al.* 2014, Patt *et al.* 2024). In this study, we approach this issue by examining how the neural representations of amount and time, two key attributes in delay discounting, are modulated by contextual valence (gains vs. losses) during outcome evaluation through the lens of neural dynamics.

The neural dynamics underlying outcome evaluation can be unfolded using the event-related potential (ERP) technique with its fine-grained temporal resolution (Glazer *et al.* 2018). Three most relevant components are the reward positivity (RewP), the P3, and the late positive potential (LPP). The RewP is a frontocentrally distributed, positive-going deflection that peaks between 250 and 350 ms following feedback onset (Proudfit 2015). It is thought to reflect reward sensitivity (Proudfit 2015) or reward prediction error (Holroyd and Coles 2002). The RewP is typically more positive for large versus small rewards (Goyer *et al.* 2008, Gu *et al.* 2011, Meadows *et al.* 2016) and less positive as the time of reward receipt increases (Cherniawsky and Holroyd 2013, Schmidt *et al.* 2017, Zheng *et al.* 2023a, Zheng *et al.* 2023b). The subsequent P3 is a parietal positivity that typically emerges between 350 and 550 ms after feedback onset (San Martin 2012). It is associated with attention allocation to salient outcome stimuli (Nieuwenhuis *et al.* 2005) and shows increased amplitude with larger reward amounts (Yeung and Sanfey 2004, Goyer *et al.* 2008, Wu and Zhou 2009, Polezzi *et al.* 2010, Gu *et al.* 2011, Meadows *et al.* 2016). Moreover, the P3 has been found to be more positive for immediate versus delayed outcomes (Zheng *et al.* 2023a, Zheng *et al.* 2023b). Directly following the P3, the LPP is a sustained positive deflection over centroparietal areas that arises approximately from 600 ms and continues onward for up to several seconds (Hajcak *et al.* 2010), reflecting the extended cognitive processing of the affective value of outcome stimuli (Glazer *et al.* 2018). Unlike the P3, the LPP is sensitive to reward amount (Pornpattananangkul and Nusslock 2015, Meadows *et al.* 2016) but not to reward time (Zheng *et al.* 2023b).

While previous studies have established that outcome-related neural dynamics are sensitive to both time and amount attributes, no research has examined how neural effects of the two attributes on outcome evaluation are influenced by contextual valence. Only a few studies have examined the effect of contextual valence on either time or amount. Regarding reward amount, several studies have observed that the RewP scales positively with reward amount in the gain context but not in the loss context (Holroyd *et al.* 2004, Sambrook *et al.* 2012, Kujawa *et al.* 2013, Zheng *et al.* 2017, Yaple *et al.* 2018). Although some findings for the P3 have revealed a similar valence-dependent pattern (Kujawa *et al.* 2013, Pfabigan *et al.* 2015, Zheng *et al.* 2023b), other studies have shown that the P3 is sensitive to outcome amount regardless of contextual valence (Zheng *et al.* 2015, Zheng *et al.* 2017). Only two studies have examined the effect of contextual valence on the processing of time attribute and found that the RewP was discounted by time delay in both gain and loss contexts (Blackburn *et al.* 2012, Mason *et al.* 2012).

To our knowledge, only one study has examined the effect of contextual valence on neural dynamics underlying amount and time (Zheng *et al.* 2023b). In this study, participants received small or large gains or losses that were delivered immediately or six months later. Results showed that in the gain context, both time and amount attributes were processed during the RewP and P3 periods in a parallel way. In contrast, in the loss context, they were processed in a serial way such that time attribute was tracked during the RewP and P3 periods, followed by amount attribute during the LPP period. However, one limitation of this study is an unbalanced presence of time and amount attributes. Specifically, time attribute was known prior to outcome delivery, whereas amount attribute was not resolved until outcome delivery. This unbalanced design might dilute neural responses to time attribute via an adaptive scaling (Walsh and Anderson 2012) but amplify neural responses to amount attribute via an unpredicted error signal (Duncan-Johnson and Donchin 1977, Hajcak *et al.* 2007). Another limitation is the reliance on dichotomous outcome delivery. Contrasting only two levels of time (e.g., today vs. 6 months later) or amount (e.g., high vs. low) may lack sensitivity in examining whether reward time and amount relate to changes in neural responses in a dose–response fashion. This is particularly important as feedback outcomes are likely encoded in a graded rather than a purely dichotomous way (Anguera *et al.* 2009, Frömer *et al.* 2016).

Here, we investigated how contextual valence affects neural dynamics underlying time and amount attributes during outcome evaluation. Participants performed a simple guessing task in two contexts: a gain context with varying reward amounts delivered at different time delays and a loss context with varying loss amounts delivered similarly. We used a parametric approach to manipulate time and amount attributes, allowing us to better track potential graded changes in neural dynamics. Based on previous findings, we hypothesized that time and amount attributes would be processed parallelly in the gain context and serially in the loss context. Specifically, the RewP and P3 would track both time and amount attributes in the gain context. In the loss context, the RewP and P3 would track time attribute, while the subsequent LPP would track amount attribute. Moreover, we explored whether these neural effects were modulated by individual differences in delay discounting, which was estimated from a classic delay discounting task (Du *et al.* 2002). Previous studies have associated the effect of time on the RewP with delay discounting but failed to take contextual valence into account (Cherniawsky and Holroyd 2013, Huang *et al.* 2017). We expected that the relationship between behavioral and neural discounting would be modulated by contextual valence.

## Materials and methods

### Participants

We recruited 40 university students (20 females; *M *= 20.38 years) for this study. All were right-handed with normal or corrected-to-normal vision acuity and free of psychiatric or neurological conditions. This sample size was determined based on previous ERP research on delay discounting (Zheng *et al.* 2023b). A sensitivity analysis was performed on each effect of interest using *simr* v1.0.6 package (Green and MacLeod 2016) to compare the regression weights with the smallest effect detectable at a power of 80% given the current sample size. Overall, these results suggest that our findings are reliable based on a sample of 40 participants (see Table S1 in the Supplementary Materials for detailed results). Each participant provided informed written consent, and the study was approved by the local Institutional Review Board.

### Procedure

Participants first performed a simple guessing task in which their electroencephalogram (EEG) data were recorded, followed by a behavioral delay discounting task. Each received a base payment of ¥10 for participation, along with a bonus from the guessing task. The EEG task lasted approximately 30 minutes and the behavioral task about 10 minutes. No other tasks or questionnaires were administered beyond demographic information.

### The guessing task

**Figure 1:**
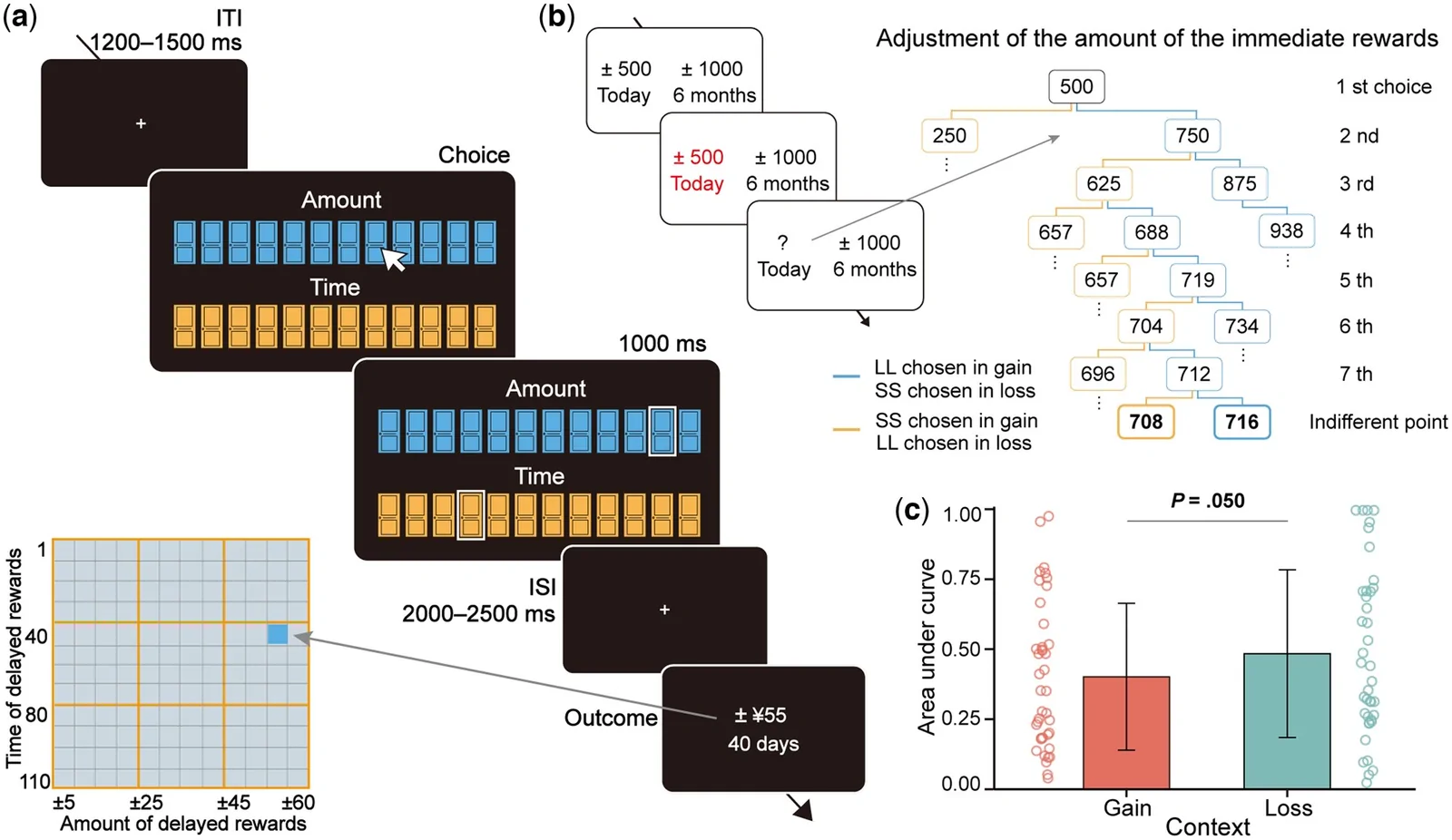
(a) The guessing task, (b) the delay discounting task, and (c) AUC values in the gain and loss contexts.

We chose the guessing task for two primary reasons. First, unlike classic delay discounting paradigm in which attribute evaluation and comparison are conflated (Kable and Glimcher 2007, Le Houcq Corbi and Soutschek 2024), the guessing task allows a more direct assessment of attribute evaluation. Second, this task ensured balanced signal–noise ratios across experimental conditions for ERP analysis because participants’ door choices did not influence the decision outcomes (see details below). The guessing task was performed in a gain context and in a loss context. In the gain context, each task trial (Fig. 1a) began with two rows of 12 doors in different colors (blue or yellow) displayed on the screen. The top row of doors corresponded to reward amounts ranging from +¥5 to +¥60 in increments of +¥5, and the bottom row of doors corresponded to the time to receive the reward, ranging from 1 to 110 days in increments of 9 or 10 days. Participants used a mouse to choose one door from the first and second rows, determining the amount and time of the reward. The selected doors were highlighted for 1000 ms, followed by a jittered interval between 2000 and 2500 ms. Subsequently, trial outcome was presented for 1500 ms, showing both the reward amount and time. Participants were encouraged to use any strategy they wanted to select the doors in order to obtain desirable outcomes. Unbeknownst to them, the outcome of each trial was predetermined and pseudorandom such that participant received one of 144 unique combinations of reward amount and time (i.e., the 12 levels of reward amount were fully crossed with the 12 levels of reward time). Each trial ended with a jittered intertrial interval between 1200 and 1500 ms. The total 144 trials of the gain context were divided into four blocks of 36 trials each, with a short break between blocks. The guessing task in the loss context was the same as in the gain context, except that participants were given an initial endowment of ¥60. Half of the participants performed the gain context first, followed by the loss context, while the other half completed the task in the reverse order. The two contexts were kept independent so that participants who performed the gain context first were unaware of the subsequent loss context, and vice versa.

Participants were instructed to consider each door choice carefully because one trial outcome from each context would be randomly selected to determine their final payment. Unbeknownst to them, the payment consisted of a gain outcome ranging from ¥30 to ¥40 with a time delay of 1 to 30 days in the gain context, and a loss outcome ranging from ¥30 to ¥20 (i.e., participants could keep an amount from ¥30 to ¥40) with a similar time delay in the loss context. Participants were required to return to the laboratory at the designated time to either receive their gain or return their loss. To reinforce the credibility of this manipulation, at the end of each context, participants recorded their final gain or loss amount and time delay on an envelope, which was then sealed and held by the experimenter until the scheduled settlement date. Before the experiment, participants were shown examples of completed envelopes from previous participants to demonstrate the procedure. Because the study was conducted on campus with registered student participants and all payments were formally documented, the settlement procedure was perceived as credible and binding. Participants were also instructed not to disclose any experiment details to other participants. After the entire experiment was completed, participants were offered the option to either return to the laboratory at the designated time to collect delayed payments or integrate all outcomes into a single final payment for immediate distribution. All participants chose the immediate option, such that no additional visit was ultimately required.

EEG data were recorded using 64 Ag/AgCl channels according to the extended 10–20 system and were referenced online to the right mastoid. Vertical electrooculogram (EOG) was recorded with a pair of channels placed below and above the left eye and horizontal EOG with a pair of channels placed at the external canthi of each eye. EEG signals were digitized at a sampling rate of 500 Hz and amplified with a Neuroscan SynAmps^2^ amplifier with a low pass of 100 Hz.

EEG data were preprocessed using EEGLAB v2021.0 (Delorme and Makeig 2004) and ERPLAB v8.10 (Lopez-Gamundi *et al.* 2021) toolboxes in MATLAB 2020b with custom routines. The data were rereferenced to the average of the left and right mastoids and filtered with a bandpass of 0.1–35 Hz using a zero phase-shift Butterworth filter (12 dB/oct roll-off). Bad channels with poor quality were interpolated from neighboring channels, and portions of EEG data with extreme voltage offsets and during break periods were removed. EEG data were submitted to an infomax independent component analysis (ICA; Delorme and Makeig 2004). Independent components related to eye blinks and movements were rejected. The ICA-corrected data were epoched from −200 to 1000 ms relative to outcome onset and baseline corrected with the activity from −200 to 0 ms. To remove additional artifacts, an automatic algorithm was applied to reject epochs with a voltage difference > 50 μV between sample points, a voltage difference > 200 μV within an epoch, or a maximum voltage difference < 0.5 μV within 100-ms intervals. This resulted in an average of 96.78% of available trials for statistical analyses.

**Figure 2:**
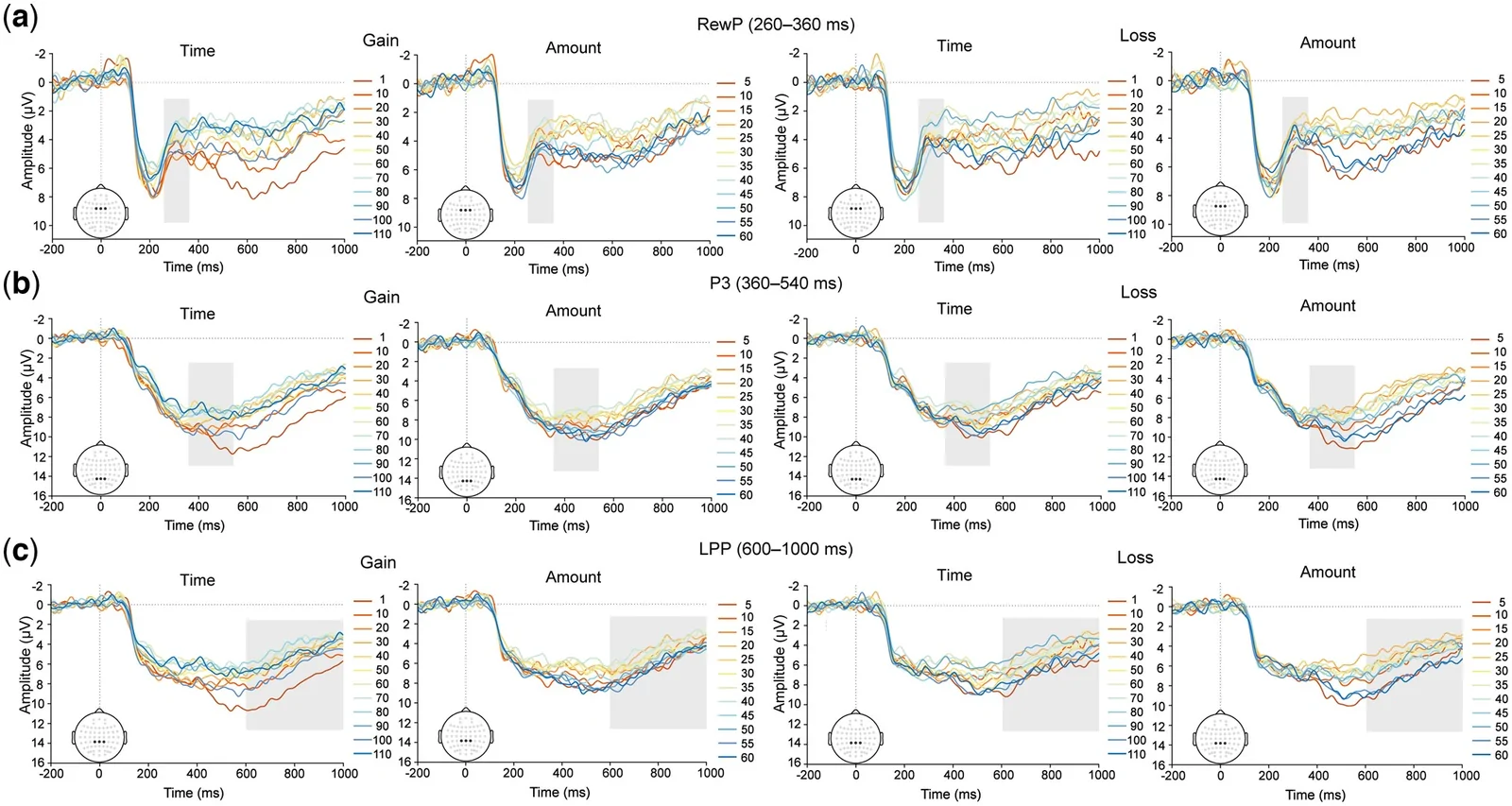
Grand-averaged ERP waveforms as a function of time and amount over frontocentral (a), parietal (b), and centroparietal (c) areas in the gain and loss contexts. Shaded vertical bars indicate time windows for quantification.

To avoid bias, measurement parameters (time windows and electrode sites) for all ERP components were defined a priori based on the established literature (Glazer *et al.* 2018), in line with best-practice recommendations (Luck and Gaspelin 2017). The grand-averaged waveforms (Fig. 2) and topographic maps (Figure S1 in the Supplementary Materials) are presented for visualization purposes. Specifically, the RewP (split-half reliability *r* = .98) was measured from 260 to 360 ms post-outcome onset over frontocentral areas (FC1, FCz, FC2); the P3 (split-half reliability *r* = .97) from 360 to 540 ms over centroparietal areas (P1, Pz, P2); and the LPP (split-half reliability *r* = .92) from 600 to 1000 ms over centroparietal areas (CP1, CPz, CP2). Each single-trial ERP dataset was fitted with linear mixed-effects regression models using the *lme4* v1.1.37 package (Bates *et al.* 2015) in R v4.4.3. Random effects structures were determined using singular value decomposition, with variables explaining zero variance being removed (Barr *et al.* 2013). Fixed effects predictors of each model included valence, time, amount, and their interactions. The categorical predictor of valence was contrast coded (−0.5 for loss and +0.5 for gain), and the continuous predictors of time and amount were standardized within participants. While our primary hypotheses focused on linear relationships, visual inspection of the data and relevant literature (e.g., Yu *et al.* 2011, Zheng *et al.* 2020) pointed toward potential nonlinear effects of outcome amount. To formally test this, we conducted post hoc exploratory analyses by adding a quadratic term for amount to the linear mixed-effects models and allowing it to interact with the predictors of context and time.

### The delay discounting task

Like the guessing task, participants performed a delay discounting task in both a gain and a loss context with hypothetical monetary amounts. In each context, participants made seven successive choices between a smaller, immediate gain/loss and a fixed, larger gain/loss of ¥1000 to be delivered at seven different times (1 week, 1 month, 6 months, 2 years, 5 years, 10 years, or 25 years). As shown in Fig. 1b, the amount of the immediate option varied across the seven choices in an iterative manner to converge on the participants’ indifference point (i.e., subjective value) for each time. Specifically, the initial choice was between a delayed gain/loss and an immediate gain/loss equal to half of the delayed amount. For the subsequent six choices, the amount of the immediate option in the gain context decreased if participants chose the immediate option but increased if they chose the delayed option. Conversely, in the loss context, the amount of the immediate option increased if participants chose the immediate option but decreased if they chose the delayed option. The change in the amount of the immediate option was always half of the previous change. The amount of the immediate option on the eighth choice was used as an estimate of the subjective value of the delayed gain/loss. To avoid choice errors, the selected option was highlighted in red, and participants could restart their choices if they were unsatisfied with any of the seven choices. Participants were informed that the money was hypothetical and needed to be imagined as real money.

To assess discounting for delayed gains and losses, we calculated the area under the discounting curve (AUC) in the gain and loss contexts separately. The AUC represents the areas under the curve formed by indifference points at each time, with values ranging from 0 to 1. A greater value indicates a stronger preference for delayed outcomes (Myerson *et al.* 2001). Given the well-known valence effect in the literature (Thaler 1981, Estle *et al.* 2006), we used a paired sample *t*-test (one-sided) to test whether the subjective value, as indexed by the AUC value, was smaller in the gain context than the loss context.

## Results

### Behavioral data

As shown in Fig. 1c, the AUC value was larger in the loss context (0.48 ± 0.30) than in the gain context (0.40 ± 0.26), *t*(39) = 1.69, *P* = .050, Cohen’*d *= 0.27, indicating a trend toward less delay discounting for losses than for gains.

### Electrophysiological data

#### Main analyses: Linear effects of time and amount

**Figure 3:**
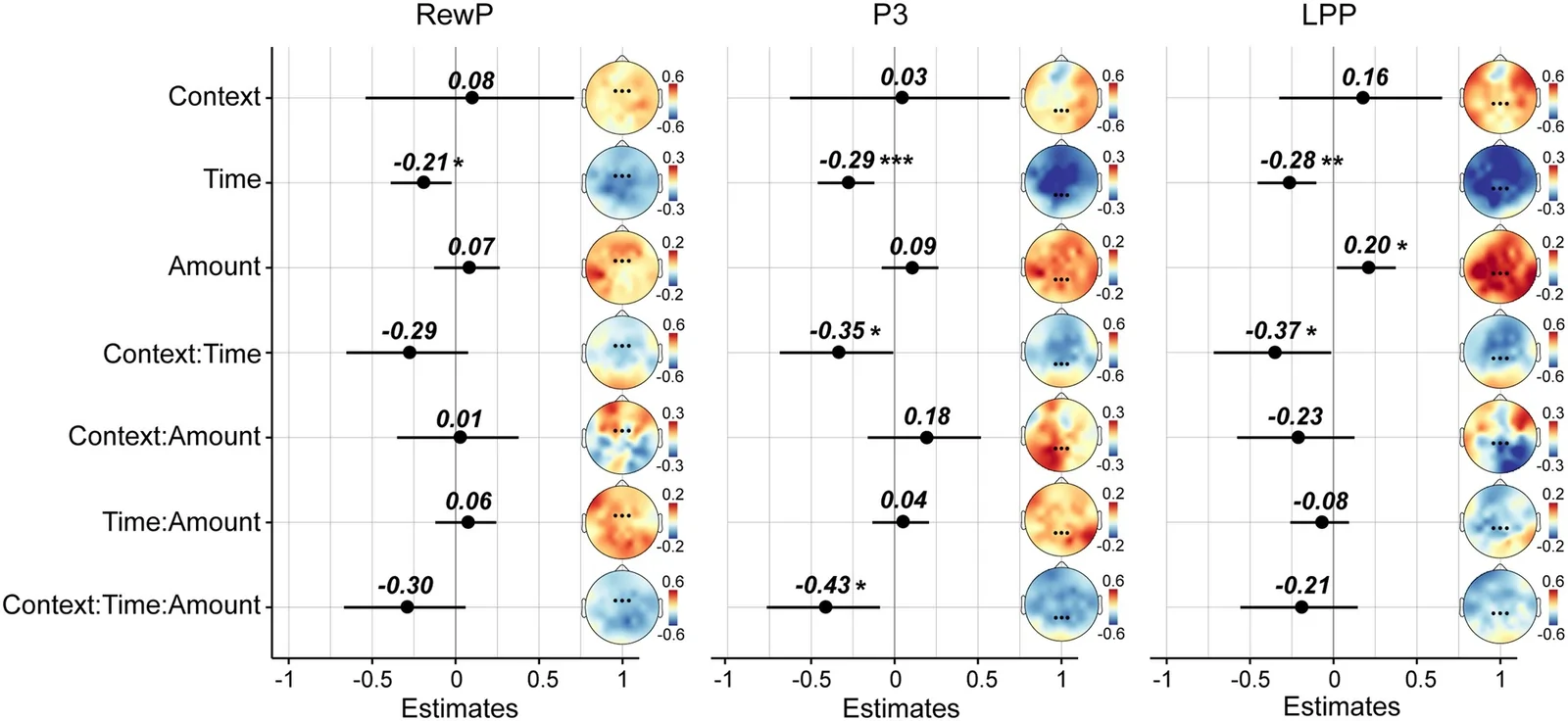
Estimated beta weights for the linear mixed-effects models. Topographic maps depict the scalp distribution of beta weights for each fixed effect within the corresponding ERP time windows. Error bars represent the 95% confidence interval based on the statistical model. *P <.05. **P <.01. ***P <.001.

**Figure 4:**
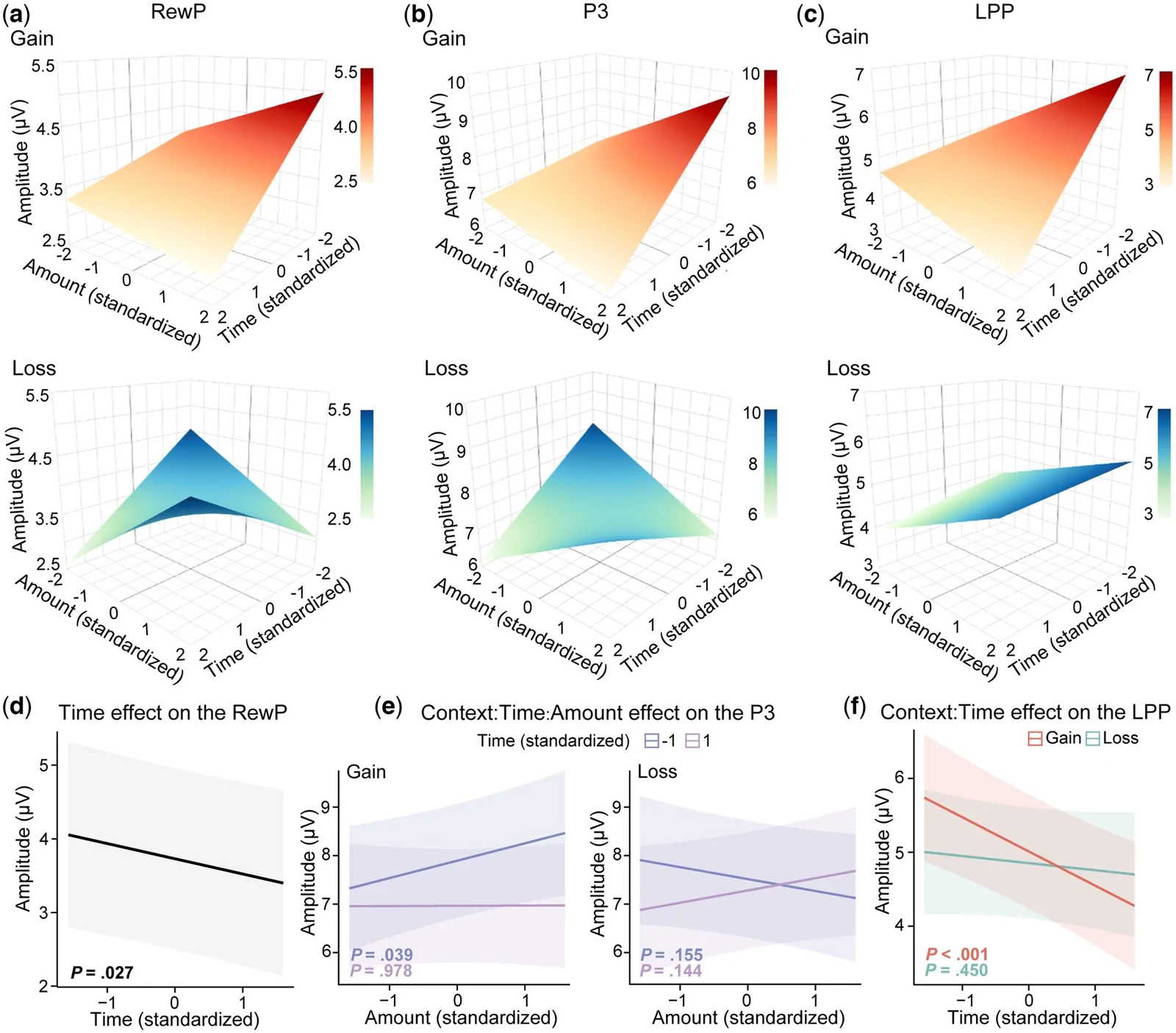
(a–c) Fixed effects of time and amount on the ERP components in the gain and loss contexts and (d–f) significant marginal effects for each component: a main effect of time on the RewP (d; predictions averaged across gain and loss at the mean amount), three-way interaction among context, time, and amount on the P3 (e), and two-way interaction between context and time on the LPP (f). Shaded areas represent the 95% confidence intervals. For visualization, the continuous predictor of time was plotted at ±1 *SD* from the mean.

Fig. 2 depicts ERP waveforms in response to time and amount attributes. Fig. 3 shows the estimated coefficients of the mixed-effects models for ERP data. Fixed effects of time and amount on the ERP components are illustrated in Fig. 4a–c. The RewP became less positive as the time delay of outcome delivery increased, *b *= −0.21, *P* = .027 (Fig. 4d), indicating a neural discounting effect. These results indicate that the RewP tracks time attribute independently of gain and loss contexts.

P3 data revealed a significant main effect of time, *b *= −0.29, *P* = .001, which was further modulated by a significant two-way interaction between context and time, *b *= −0.35, *P* = .045. Follow-up simple slopes analyses showed that the P3 became less positive as the time delay increased in the gain context, *b *= −0.47, *z* = −3.80, *P* < .001, but not the loss context, *b *= −0.12, *z* = −0.95, *P* = .340. Importantly, we observed a significant three-way interaction among context, time, and amount, *b *= −0.43, *P* = .014. As shown in Fig. 4e, the P3 in the gain context became more positive as the amount of the outcome increased when it was delayed to a smaller extent (*M − *1SD of reward time), *b *= 0.36, *z* = 2.07, *P* = .039, but not when it was delayed to a greater extent (*M *+ 1SD of reward time), *b *< 0.01, *z* = 0.03, *P* = .978. In the loss context, the P3 was not affected by the amount of the outcome, regardless of when it was delivered (*M* *−* 1SD: *b *= −0.25, *z* = −1.42, *P* = .155; *M *+ 1*SD: b *= 0.25, *z* = 1.46, *P* = .144). These results suggest that the P3 tracks time attribute in the gain context but not in the loss context, and larger rewards amplify the P3 only when they are delivered sooner in the gain context.

LPP data revealed that it became more positive as the amount of the outcome increased, *b *= 0.20, *P* = .029, and less positive as the time to receive the outcome increased, *b *= −0.28, *P* = .002. Importantly, the time effect on the LPP was observed in the gain context, *b *= −0.46, *z* = −3.65, *P* < .001, but not in the loss context, *b *= −0.10, *z* = −0.76, *P* = .450, as revealed by a significant interaction between context and time (Fig. 4f), *b *= −0.37, *P* = .041. These results suggest that time and amount attributes are tracked by the LPP in the gain context, whereas only amount attribute is tracked in the loss context.

#### Exploratory analyses: nonlinear effects of amount

To further explore potential nonlinear effects of amount, we constructed supplementary models that included the quadratic term of amount and its interactions with context and time (see Table S2 in the Supplementary Materials for detailed results). Results showed significant quadratic effects of amount for the RewP, *b *= 0.36, *P* = .002, P3, *b *= 0.79, *P* < .001, and LPP, *b *= 0.68, *P* < .001, indicating a U-shaped pattern where ERP amplitudes were larger for extreme amounts (both smallest and largest) compared to intermediate amounts. Furthermore, for the RewP and LPP, the quadratic amount effect significantly interacted with time. Simple slopes analyses revealed that for the RewP, the quadratic amount effect was significant when the outcome was delayed to a greater extent (*M *+ 1*SD: b *= 1.24, *z *= 3.93, *P* < .001), but not when it was delayed to a smaller extent (*M −* 1*SD: b *= 0.19, *z *= 0.61, *P* = .544). For the LPP, the quadratic amount effect was significant at both short (*M − *1*SD: b *= 0.82, *z *= 2.20, *P* = .028) and long delays (*M *+ 1*SD: b *= 1.89, *z *= 5.08, *P* < .001) but was significantly stronger for long delays compared to short delays, *b *= 1.07, *z *= 2.65, *P* = .008. Crucially, the inclusion of the quadratic term did not qualitatively alter the significance or direction of the primary linear effects of time and amount reported above, except that the context-by-time interaction for the P3 and LPP became nonsignificant. However, the higher-order three-way interaction among context, time, and amount on the P3 remained significant, *b *= −0.43, *P* = .012, reinforcing that the integration of time and amount differed by valence. Regarding the LPP, although the context-by-time interaction did not reach statistical significance in the quadratic model, simple slopes analyses indicated that the time effect remained significant in the gain context, *b *= −0.75, *z *= −3.93, *P* < .001, but not in the loss context, *b *= −0.34, *z *= −1.78, *P* = .075, a pattern consistent with the primary linear analysis.

### Individual difference of delay discounting on neural dynamics

To investigate how neural dynamics underlying amount and time attributes are modulated by individual difference in delay discounting, we constructed a series of linear mixed-effects regression models for our ERP data, including predictors of time, amount, AUC values (standardized across participants) estimated from the delay discounting task, and their interactions, separately for the gain and loss contexts. Detailed model results are shown in Table S3–S5 in the Supplementary Materials.

For each ERP component (Figure S2 in the Supplementary Materials), we observed a significant three-way interaction among time, amount, and AUC value in the loss context (RewP: *b *= −0.31, *P* = .012; P3: *b *= −0.32, *P* = .006; LPP: *b *= −0.32, *P* = .009). For high discounters, both the RewP and P3 became less positive as outcome amount increased when it was delayed to a smaller extent (*M − *1*SD* of reward time, RewP: *b *= −0.60, *z* = −2.19, *P* = .028; P3: *b *= −0.68, *z* = −2.50, *P* = .014) but more positive as outcome amount increased when it was delayed to a larger extent (*M *+ 1*SD* of reward time, RewP: *b *= 0.54, z = 1.96, *P* = .050; P3: *b *= 0.56, *z* = 2.04, *P* = .042). For low discounters, both the RewP and P3 were not affected by outcome amount, regardless of whether it was delivered earlier (RewP: *b *= 0.19, *z* = 0.79, *P* = .432; P3: *b *= 0.08, *z* = 0.33, *P* = .744) or later (RewP: *b *= 0.08, *z* = 0.32, *P* = .751; P3: *b *= 0.02, *z* = 0.09, *P* = .930). For high discounters, the LPP became more positive as outcome amount increased when it was delayed to a larger extent (*M *+ 1*SD*), *b *= 0.66, *z* = 2.49, *P* = .013, but not when it was delayed to a smaller extent (*M −* 1*SD*), *b *= −0.10, *z* = −0.38, *P* = .705. For low discounters, the LPP became more positive as outcome amount increased when it was delayed to a smaller extent (*M − *1*SD*), *b *= 0.58, *z* = 2.52, *P* = .012, but not when it was delayed to a larger extent (*M *+ 1*SD*), *b *= 0.08, *z* = 0.35, *P* = .729. In contrast, no significant effects involving AUC were found for the gain context. Together, these exploratory results show that individual differences in behavioral delay discounting are associated with neural responses in the loss context but not in the gain context.

## Discussion

In this study, we investigated how contextual valence affects neural dynamics underlying time and amount, two key attributes in delay discounting, during outcome evaluation. We manipulated these attributes in a balanced, parametric manner to better track their neural variations. As expected, neural processing of time and amount attributes showed different neural patterns between gain and loss contexts. In the gain context, time was first tracked during the RewP period, then time and linearly increasing monetary value were integrated during the P3 period, and finally processed independently during the LPP period. In the loss context, while time was similarly processed during the RewP period, monetary value was not tracked until the LPP period. Additionally, individual differences in delay discounting were associated with neural responses in the loss context but not the gain context. Our findings suggest asymmetrical neural patterns between gain and loss contexts.

The RewP became less positive as the time until outcome delivery increased, regardless of whether it was a gain or a loss. Considering the RewP a reliable neural signal for reward sensitivity (Proudfit 2015), our finding of the time effect on the RewP aligns with the well-known delay discounting phenomenon (Green and Myerson 2004). Importantly, the neural discounting effect was comparable between gain and loss contexts, suggesting that gains and losses are discounted at a similar rate during the RewP period (Blackburn *et al.* 2012, Mason *et al.* 2012, Zheng *et al.* 2023b). Moreover, the RewP was insensitive to linear variations in outcome amount across gain and loss contexts. These results support the independent coding hypothesis of the RewP (Yeung and Sanfey 2004). According to this theory, our brain processes valence and amount separately: we first evaluate the valence dimension and then process the amount dimension. The RewP is thought to reflect the early binary evaluation of good versus bad outcomes (Hajcak *et al.* 2006) and the most salient information during outcome evaluation (Nieuwenhuis *et al.* 2004). In our task context, when presented time and amount attributes of gains and losses, participants first processed how long the gain/loss would be delivered rather than its monetary value. The RewP may reflect this initial evaluation of the time attribute to determine how good or bad the outcomes are. After this initial evaluation, monetary value was incorporated to scale the value of the outcomes, as indexed by the P3 and LPP. It should be noted that our finding contradicts several previous studies observing that the RewP is sensitive to outcome amount (Goyer *et al.* 2008, Gu *et al.* 2011, Zheng *et al.* 2023b). These discrepancies are attributable to the conflation of reward valence and amount in these studies, which included only two levels of amount attribute (e.g., high vs. low). Therefore, the observed amount effect in previous research may actually be a valence effect because reward processing adjusts to the range of possible outcomes within a task context to identify a large reward as positive and a small reward as negative (Holroyd *et al.* 2004). By parameterizing outcome attributes, we teased apart neural processes of time and amount attributes.

Unlike the RewP, we observed asymmetrical neural responses to time and amount attributes between gain and loss contexts during the later periods of the P3 and LPP. In the gain context, the P3 tracked the interaction between time and amount such that the effect of monetary value on the P3 was diminished as the time delay increased. This pattern suggests that monetary value is integrated into value computation, which is likely modulated by attentional resources (Lim *et al.* 2011). Following the P3, the LPP became more positive as monetary value increased but less positive as time delay increased. The absence of the time-by-amount interaction on the LPP suggests that these attributes are processed simultaneously but independently during later stage of outcome evaluation. Together, these results suggest a temporal progression in the gain context: early integrative processing of monetary value during the P3 shifts to a more orthogonal, attribute-specific representation during the LPP. Despite their surface similarities, the P3 and LPP differ in functional correlates during outcome evaluation (San Martin 2012). The P3 reflects attentional allocation to motivationally salient stimuli (Nieuwenhuis *et al.* 2005), whereas the LPP indexes sustained cognitive and affective evaluation of outcome value (Glazer *et al.* 2018). Consistent with this distinction, our findings suggest that processing of time and amount attributes in the gain context shifts from attentional integration to affective elaboration. In contrast, in the loss context, only the LPP tracked monetary value, becoming more positive as loss amount increased, whereas neither the P3 nor the LPP was sensitive to time attribute. These blunted effects of time and amount are congruent with previous findings of reduced outcome-related P3 in aversion scenarios (Kujawa *et al.* 2013, Pfabigan *et al.* 2015, Zheng *et al.* 2023b). Together, the contrasting neural patterns across gain and loss contexts highlight how attention and emotion jointly shape the encoding of time and amount attributes in intertemporal choices. Gains engage both early attentional and later affective systems to evaluate outcome attributes comprehensively, while losses primarily elicit affective responses to monetary value with minimal attentional engagement. This imbalance aligns with theories proposing that negative emotions heighten sensitivity to losses (Camerer 2005, Tom *et al.* 2007) but may also constrain attentional processing (Zheng *et al.* 2023b). The combination of heightened affect and reduced attention may therefore contribute to the blunted discounting commonly observed in loss contexts (Thaler 1981, Estle *et al.* 2006).

Together, our ERP results revealed asymmetrical neural dynamics underlying time and amount processing between gain and loss contexts. This neural asymmetry unfolded over time, highlighting both common and distinct attribute processing between gain and loss contexts during delay discounting. For delayed gains, time attribute is tracked, then integrated with monetary value, and finally dissociated from it. For delayed losses, however, time attribute is tracked first, with monetary value processed much later in the information processing stream, showing as a serial manner. This temporal evolution of option evaluation is consistent with previous evidence suggesting that individuals tended to evaluate time attribute before considering amount attribute when making intertemporal choices (Durschmid *et al.* 2021). Importantly, our finding of distinct evaluation processes for time and amount between gains and losses sheds new light on intertemporal choices (Amasino *et al.* 2019). Classical intertemporal choice models such as hyperbolic discounting posit that reward time and amount are integrated within each option prior to comparison (Samuelson 1937, Ainslie 1975). In contrast, other theories emphasize that intertemporal choice reflects attribute-based processes in which time and amount attributes are compared separately (Roelofsma and Read 2000, Dai and Busemeyer 2014). This controversy is partially reconciled by our ERP findings. In the gain context, time and amount attributes are integrated, supporting the classical intertemporal choice models. In the loss context, time and amount attributes are processed separately, supporting the attribute-wise models. Our finding of asymmetrical neural patterns between gain and loss contexts also provides insights into a growing literature examining the gain–loss asymmetry in delay discounting (Tanaka *et al.* 2014, Molouki *et al.* 2019, Patt *et al.* 2024). Behavioral evidence has shown that people typically discount delayed gains more than delayed losses (Thaler 1981, Estle *et al.* 2006). Neuroimaging studies have suggested asymmetric neural circuits dedicated to delayed gains and losses (Xu *et al.* 2009, Zhang *et al.* 2018). Our ERP findings add a crucial and novel piece to this issue by showing distinct neural dynamics underlying time and amount attributes during outcome evaluation.

Despite its exploratory nature, individual difference analyses further support the gain–loss asymmetry in delay discounting. We found that higher discounters exhibited greater neural variation in response to time and monetary value, especially during the RewP and P3 periods, in the loss rather than the gain context. While the ERPs in our guessing task index the evaluative processing of time and monetary value, the AUC from the behavioral task reflects a more deliberative, comparative decision-making processing. The presence of correlations for losses, but not for gains, suggests that these two processes, evaluation and choice, are more tightly coupled in the loss domain. This may be a direct consequence of loss aversion, where the greater utility and motivational salience assigned to losses (Kahneman and Tversky 2000) forge a link between attribute evaluation and subsequent choice behavior (Canessa *et al.* 2013). The absence of this correlation for gains implies that these processes are less tightly coupled for rewards. However, several methodological factors may have also contributed to these findings. The behavioral delay discounting task used hypothetical outcomes and included extremely long delays (up to 25 years), which may have reduced ecological validity and participant engagement. Such design features could partially explain the marginally significant sign effect observed behaviorally and may have weakened the correspondence between discounting behavior and neural responses. Indeed, prior research has demonstrated that individuals often respond differently when choices involve tangible rather than hypothetical consequences (Hinvest and Anderson 2010). Finally, the relatively small sample size further limits statistical power, and thus these results should be interpreted as preliminary but informative, offering useful directions for future studies with larger samples and incentive-compatible designs.

Unlike the gain–loss asymmetry observed in the linear valuation process, our exploratory analyses revealed a consistent U-shaped quadratic relationship between outcome amount and neural responses (the RewP, P3, and LPP) across both the gain and loss context, where amplitudes were larger for extreme amounts (low and high) compared to intermediate ones. This finding suggests that in addition to tracking value (linear), the brain also tracks outcome saliency or expectancy violation (nonlinear). In our design with uniform probability across 12 amounts, participants may have formed an implicit expectation of a mean outcome. Extreme values, being furthest from this expectation, likely generated stronger saliency signals. This aligns with the view that the P3/LPP reflects context updating and attention to informative stimuli (Donchin 1981). Notably, for the RewP and LPP, this saliency signal was modulated by time, becoming stronger as delay increased. This interaction aligns with construal level theory (Trope and Liberman 2003), which posits that individuals construe near-future events in terms of concrete, low-level details, whereas distant-future events are represented by abstract, high-level features. In the context of outcome evaluation, the precise monetary value (tracking linearly) represents a concrete detail that dominates processing when outcomes are immediate. As temporal distance increases, the neural system may shift toward processing the abstract “gist” or motivational salience of the outcome, specifically its extremity (tracking quadratically), to maintain the distinctiveness of significant consequences despite value discounting. However, given the exploratory nature of our results, future studies are needed to replicate these findings and further elucidate how temporal distance modulates the neural dynamics of outcome value versus saliency.

One caveat of the present study is that, although participants were informed that monetary outcomes of the guessing task would be delivered at varying delays, all payments were made immediately after the experiment. Although participants were instructed not to disclose experimental details to other participants, this manipulation may still limit the validity of the task if some participants learned (e.g., from gossip among other participants) that payments were not actually delayed. Nonetheless, the significant correlations between ERP responses and behavioral discounting suggest that most participants engaged with the task as intended. Future studies should consider implementing real delayed payments or post-task credibility checks.

In conclusion, this study offers a novel perspective on the gain–loss asymmetry in delay discounting through neural dynamics underlying time and amount evaluation. For gains, time attribute was first tracked during the RewP period, then integrated with monetary value during the P3 period, and finally processed independently from monetary value during the LPP period. For losses, time attribute was processed during the RewP period, but monetary value was not tracked until the LPP period. Given that gain–loss asymmetry is linked to various social issues such as smoking (Odum *et al.* 2002) as well as debt and obesity (Ikeda *et al.* 2010), our findings may contribute to understanding the mechanisms underlying these maladaptive behaviors.

## Supplementary Material

nsag029_Supplementary_Data

## Data Availability

Data and code that support the findings of this study are available at https://osf.io/pkeb2/.
