# Supplementary material for: Time and amount attributes exert distinct neural influences on gain and loss evaluation
Source: Soc Cogn Affect Neurosci. 2026 May 6;21(1):nsag029. doi: 10.1093/scan/nsag029 (PMC13200790; doi:10.1093/scan/nsag029)
Supplement: nsag029_Supplementary_Data [file nsag029_supplementary_data.docx]

**Supplementary Materials**

**Table S1.** Results from the sensitivity analysis

| Regression equation | Tested effect | *P* | Original effect  (*b* ± *SE*) | Smallest effect  (*b*) |
| --- | --- | --- | --- | --- |
| RewP ~ Context × Time × Amount | Time | .027 | -0.21 ± 0.09 | -0.26 |
| P3 ~ Context × Time × Amount | Time | .001 | -0.29 ± 0.09 | -0.25 |
|  | Context:Time | .045 | -0.35 ± 0.17 | -0.48 |
|  | Context:Time:Amount | .014 | -0.43 ± 0.17 | -0.47 |
| LPP ~ Context × Time × Amount | Time | .002 | -0.28 ± 0.09 | -0.25 |
|  | Amount | .029 | 0.20 ± 0.09 | 0.25 |
|  | Context:Time | .041 | -0.37 ± 0.18 | -0.49 |

*Notes.* Smallest effect size refers to the smallest detectable *b* at 80% power. For simplicity, only the fixed effect structure is displayed in the column detailing regression equations. *SE* = standard error.


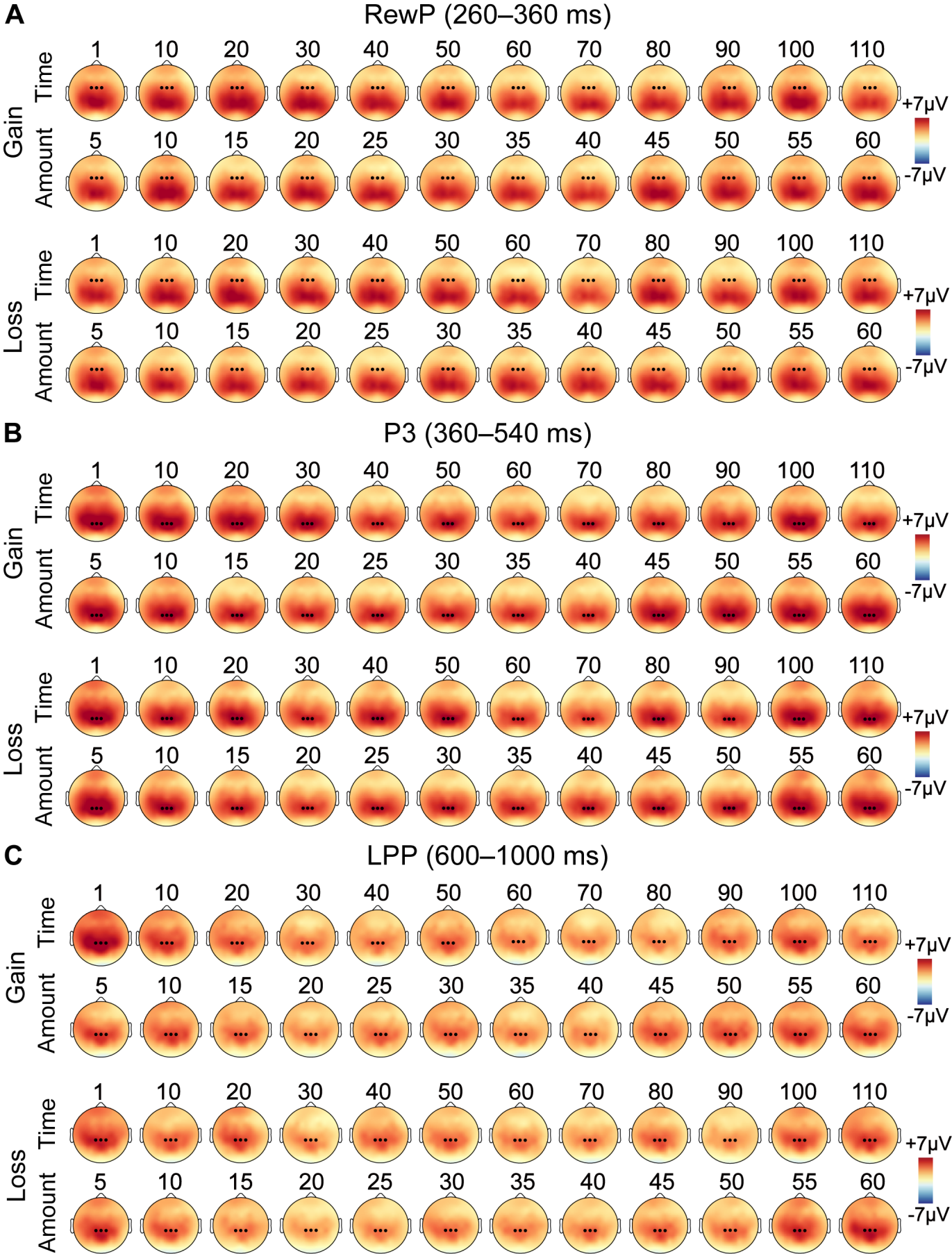


**Figure S1.** Topographic maps for the RewP (A), P3 (B), and LPP (C) as a function of time and amount in the gain and loss contexts.

**Table S2.** Model results of the linear mixed-effects regression including the quadratic term of amount

|  | **RewP** | | |  | **P3** | | |  | **LPP** | | |
| --- | --- | --- | --- | --- | --- | --- | --- | --- | --- | --- | --- |
| Predictors | *b* | 95% CI | *P* |  | *b* | 95% CI | *P* |  | *b* | 95% CI | *P* |
| Intercept | 3.37 | 2.16, 4.59 | **< .001** |  | 6.63 | 5.52, 7.73 | **< .001** |  | 4.22 | 3.52, 4.92 | **< .001** |
| Context (C) | -0.06 | -0.81, 0.69 | .870 |  | 0.12 | -0.64, 0.88 | .762 |  | 0.44 | -0.19, 1.07 | .171 |
| Time (T) | -0.47 | -0.74, -0.19 | **.001** |  | -0.43 | -0.69, -0.18 | **.001** |  | -0.55 | -0.81, -0.28 | **< .001** |
| Amount (A) | 0.07 | -0.11, 0.25 | .468 |  | 0.09 | -0.12, 0.30 | .391 |  | 0.19 | 0.02, 0.37 | **.030** |
| Amount^2^ (A^2^) | 0.36 | 0.13, 0.59 | **.002** |  | 0.79 | 0.49, 1.08 | **< .001** |  | 0.68 | 0.37, 0.98 | **< .001** |
| C:T | -0.12 | -0.67, 0.43 | .665 |  | -0.19 | -0.70, 0.32 | .460 |  | -0.41 | -0.94, 0.12 | .127 |
| C:A | 0.01 | -0.35, 0.37 | .955 |  | 0.18 | -0.16, 0.52 | .300 |  | -0.23 | -0.58, 0.12 | .201 |
| C:A^2^ | 0.15 | -0.27, 0.56 | .486 |  | -0.09 | -0.47, 0.30 | .657 |  | -0.28 | -0.68, 0.11 | .163 |
| T:A | 0.06 | -0.12, 0.24 | .512 |  | 0.04 | -0.13, 0.21 | .652 |  | -0.08 | -0.26, 0.09 | .360 |
| T:A^2^ | 0.26 | 0.06, 0.47 | **.013** |  | 0.14 | -0.05, 0.33 | .143 |  | 0.27 | 0.07, 0.47 | **.008** |
| C:T:A | -0.30 | -0.67, 0.06 | .101 |  | -0.43 | -0.77, -0.10 | **.012** |  | -0.21 | -0.56, 0.14 | .231 |
| C:T:A^2^ | -0.17 | -0.58, 0.24 | .422 |  | -0.16 | -0.54, 0.23 | .425 |  | 0.04 | -0.35, 0.44 | .834 |

*Note.* The final model is described using Wilkinson notation as: Amplitude ~ Context * Time * (Amount + Amount^2^) + (Context + Amount^2^ | Participant) for the RewP and LPP, and Amplitude ~ Context * Time * (Amount + Amount^2^) + (Context + Amount^2^ + Amount | Participant) for the P3. Statistically significant *P* values (< .05, two-sided) are shown in bold. CI = confidence interval.

**Table S3.** Model results of the linear mixed-effects regression for the RewP in the gain and loss contexts.

|  | **Gain context** | | |  | **Loss context** | | |
| --- | --- | --- | --- | --- | --- | --- | --- |
| Predictors | *b* | 95% CI | *P* |  | *b* | 95% CI | *P* |
| Intercept | 3.72 | 2.47, 4.96 | **< .001** |  | 3.62 | 2.32, 4.91 | **< .001** |
| Time | -0.35 | -0.61, -0.09 | **.009** |  | -0.03 | -0.29, 0.23 | .803 |
| Amount | 0.08 | -0.19, 0.34 | .569 |  | 0.05 | -0.21, 0.31 | .701 |
| AUC | -0.38 | -1.72, 0.95 | .573 |  | 0.48 | -0.75, 1.70 | .445 |
| Time:Amount | -0.08 | -0.34, 0.18 | .541 |  | 0.26 | -0.00, 0.52 | .052 |
| Time:AUC | 0.01 | -0.27, 0.29 | .950 |  | -0.18 | -0.43, 0.06 | .143 |
| Amount:AUC | 0.02 | -0.26, 0.31 | .883 |  | 0.08 | -0.16, 0.33 | .513 |
| Time:Amount:AUC | 0.07 | -0.21, 0.35 | .614 |  | -0.31 | -0.56, -0.07 | **.012** |

*Note.* The final model is described using Wilkinson notation as: Amplitude ~ Time * Amount * AUC + (Amount | Participant) in the gain context and Amplitude ~ Time * Amount * AUC + (1 | Participant) in the loss context. Statistically significant *P* values (< .05, two-sided) are shown in bold. CI = confidence interval; AUC = area under the discounting curve.

**Table S4.** Model results of the linear mixed-effects regression for the P3 in the gain and loss contexts.

|  | **Gain context** | | |  | **Loss context** | | |
| --- | --- | --- | --- | --- | --- | --- | --- |
| Predictors | *b* | 95% CI | *P* |  | *b* | 95% CI | *P* |
| Intercept | 7.39 | 6.23, 8.54 | **< .001** |  | 7.29 | 6.12, 8.45 | **< .001** |
| Time | -0.47 | -0.72, -0.23 | **< .001** |  | -0.10 | -0.35, 0.14 | .402 |
| Amount | 0.16 | -0.13, 0.44 | .281 |  | -0.00 | -0.28, 0.27 | .975 |
| AUC | -0.30 | -1.54, 0.94 | .638 |  | 0.75 | -0.35, 1.85 | .181 |
| Time:Amount | -0.15 | -0.39, 0.09 | .227 |  | 0.30 | 0.05, 0.54 | **.017** |
| Time:AUC | -0.06 | -0.32, 0.21 | .680 |  | -0.08 | -0.31, 0.15 | .473 |
| Amount:AUC | -0.16 | -0.46, 0.15 | .311 |  | 0.05 | -0.21, 0.32 | .683 |
| Time:Amount:AUC | 0.20 | -0.06, 0.46 | .134 |  | -0.32 | -0.55, -0.09 | **.006** |

*Note.* The final model is described using Wilkinson notation as: Amplitude ~ Time * Amount * AUC + (Amount + Time | Participant) in the gain context and Amplitude ~ Time * Amount *AUC + (Amount | Participant) in the loss context. Statistically significant *P* values (< .05, two-sided) are shown in bold. CI = confidence interval; AUC = area under the discounting curve.

**Table S5.** Model results of the linear mixed-effects regression for the LPP in the gain and loss contexts.

|  | **Gain context** | | |  | **Loss context** | | |
| --- | --- | --- | --- | --- | --- | --- | --- |
| Predictors | *b* | 95% CI | *P* |  | *b* | 95% CI | *P* |
| Intercept | 4.93 | 4.15, 5.70 | **< .001** |  | 4.74 | 4.00, 5.48 | **< .001** |
| Time | -0.45 | -0.70, -0.20 | **< .001** |  | -0.09 | -0.34, 0.16 | .487 |
| Amount | 0.05 | -0.21, 0.30 | .723 |  | 0.31 | 0.06, 0.56 | **.017** |
| AUC | -0.28 | -1.11, 0.55 | .514 |  | 0.47 | -0.23, 1.16 | .190 |
| Time:Amount | -0.16 | -0.41, 0.09 | .218 |  | 0.06 | -0.19, 0.32 | .615 |
| Time:AUC | 0.07 | -0.20, 0.34 | .599 |  | -0.04 | -0.27, 0.20 | .771 |
| Amount:AUC | -0.25 | -0.52, 0.02 | .066 |  | 0.03 | -0.21, 0.26 | .831 |
| Time:Amount:AUC | 0.19 | -0.08, 0.46 | .168 |  | -0.32 | -0.55, -0.08 | **.009** |

*Note.* The final model is described using Wilkinson notation as: Amplitude ~ Time * Amount * AUC + (1 | Participant) in the gain and loss contexts. Statistically significant *P* values (< .05, two-sided) are shown in bold. CI = confidence interval; AUC = area under the discounting curve.


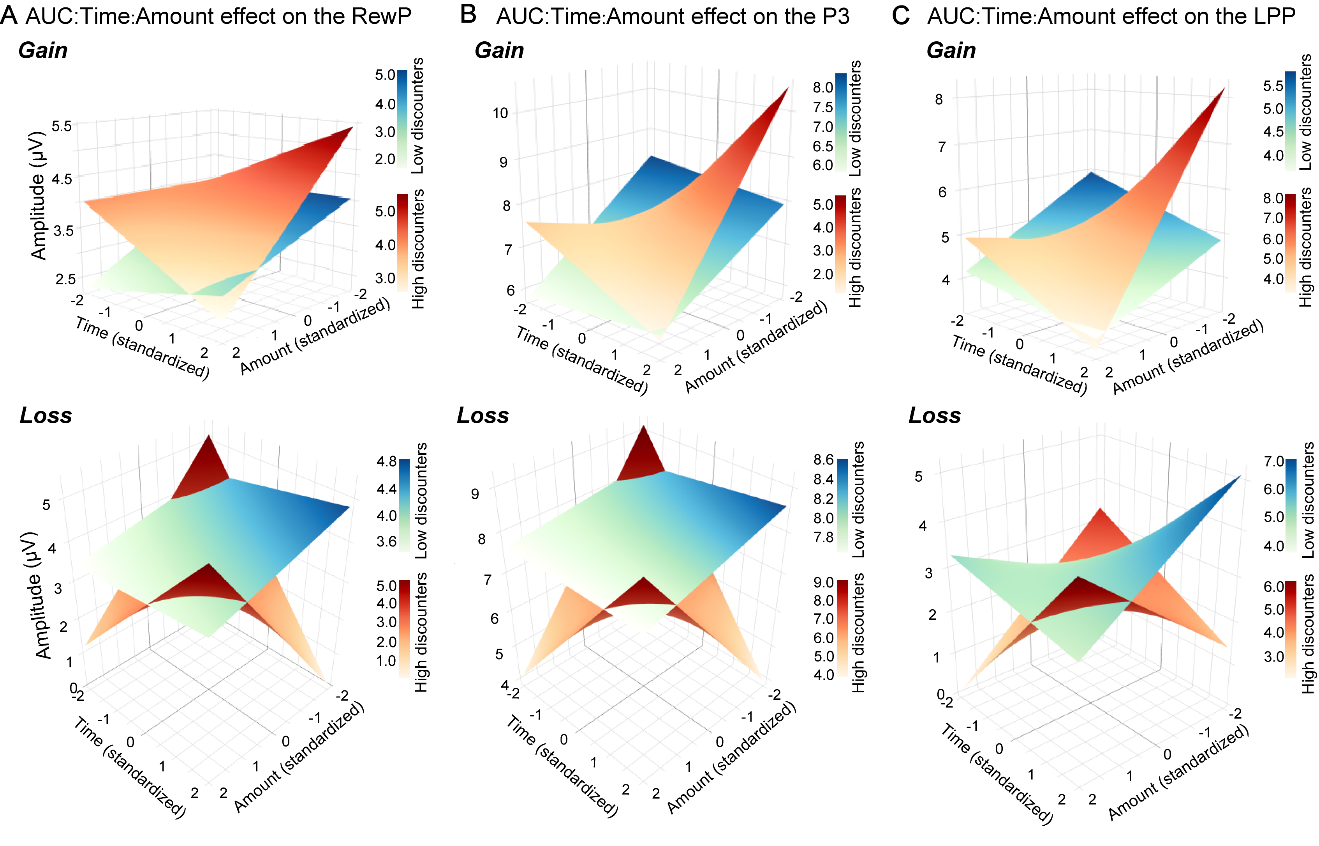


**Figure S2.** Fixed effects of time and amount for high discounters (*M* – 1*SD* of AUC value) and low discounters (*M* + 1*SD* of AUC value) on the RewP (A), P3 (B), and LPP (C) in the gain (top) and loss (bottom) contexts.
